# Species composition, seasonal occurrence, habitat preference and altitudinal distribution of malaria and other disease vectors in eastern Nepal

**DOI:** 10.1186/s13071-014-0540-4

**Published:** 2014-11-28

**Authors:** Meghnath Dhimal, Bodo Ahrens, Ulrich Kuch

**Affiliations:** Nepal Health Research Council (NHRC), Ministry of Health and Population Complex, Ramshah Path, Kathmandu, Nepal; Biodiversity and Climate Research Centre (BiK-F), Senckenberg Gesellschaft für Naturforschung, Frankfurt am, Germany; Institute for Atmospheric and Environmental Sciences (IAU), Goethe University, Frankfurt am Main, Germany; Institute of Occupational Medicine, Social Medicine and Environmental Medicine, Goethe University, Frankfurt am Main, Germany

**Keywords:** *Aedes*, *Anopheles*, *Culex*, Climate change, Dengue, Elimination, Japanese encephalitis, Lymphatic filariasis, Mosquito-borne diseases

## Abstract

**Background:**

It is increasingly recognized that climate change can alter the geographical distribution of vector-borne diseases (VBDs) with shifts of disease vectors to higher altitudes and latitudes. In particular, an increasing risk of malaria and dengue fever epidemics in tropical highlands and temperate regions has been predicted in different climate change scenarios. The aim of this paper is to expand the current knowledge on the seasonal occurrence and altitudinal distribution of malaria and other disease vectors in eastern Nepal.

**Methods:**

Adult mosquitoes resting indoors and outdoors were collected using CDC light trap and aspirators with the support of flash light. Mosquito larvae were collected using locally constructed dippers. We assessed the local residents’ perceptions of the distribution and occurrence of mosquitoes using key informant interview techniques. Generalized linear models were fitted to assess the effect of season, resting site and topography on the abundance of malaria vectors.

**Results:**

The known malaria vectors in Nepal, *Anopheles fluviatilis*, *Anopheles annularis* and *Anopheles maculatus* complex members were recorded from 70 to 1,820 m above sea level (asl). The vectors of chikungunya and dengue virus, *Aedes aegypti* and *Aedes albopictus*, the vector of lymphatic filariasis, *Culex quinquefasciatus*, and that of Japanese encephalitis, *Culex tritaeniorhynchus*, were found from 70 to 2,000 m asl in eastern Nepal. Larvae of *Anopheles*, *Culex* and *Aedes* species were recorded up to 2,310 m asl. Only season had a significant effect on the abundance of *An. fluviatilis*, season and resting site on the abundance of *An. maculatus* complex members, and season, resting site and topography on the abundance of *An. annularis*. The perceptions of people on mosquito occurrence are consistent with entomological findings.

**Conclusions:**

This study provides the first vertical distribution records of vector mosquitoes in eastern Nepal and suggests that the vectors of malaria and other diseases have already established populations in the highlands due to climatic and other environmental changes. As VBD control programmes have not been focused on the highlands of Nepal, these findings call for actions to start monitoring, surveillance and research on VBDs in these previously disease-free, densely populated and economically important regions.

## Background

Malaria, transmitted by the bite of infected *Anopheles* mosquitoes, also known as malaria vectors, is the oldest reported tropical disease in Nepal. The first efforts to survey the mosquitoes and identify the vectors of malaria in Nepal were initiated in 1952 [1]. So far, 44 species of *Anopheles* mosquitoes have been identified in Nepal based on morphological characteristics but only seven have been reported as malaria vectors of primary importance [2,3]. These include: *Anopheles minimus, Anopheles fluviatilis, Anopheles annularis*, *Anopheles maculatus, Anopheles dravidicus, Anopheles pseudowillmori*, and *Anopheles willmori* [3]. The last four species are commonly reported as ‘*Anopheles maculatus* complex members’ in Nepal. It has been reported that deforestation and effective control using DDT practically eliminated *An. minimus* in Nepal during the 1960s [4], and *An. fluviatilis* is now the primary malaria vector in Nepal, *An. annularis* the secondary malaria vector and the *An. maculatus* complex members are seasonal malaria vectors in the mountain region of Nepal [2-6]. However, in absence of molecular tools at that time, the reported selective elimination of *An. minimus* from Nepal might have reflected taxonomic uncertainty with respect to morphologically cryptic mosquito species rather than a real elimination from the country [7,8]. Other *Anopheles* species from the group of 44 have been incriminated elsewhere in Asia [9-11] and may play a role in malaria transmission under changing environments and climate in Nepal [12].

Entomological surveys of malaria vectors are very important for controlling vector populations and diseases in the community. However, little attention has been given to entomological surveillance and vector control in Nepal [4,6,13,14]. In addition, a government effort to control malaria is further threatened by insecticide and drug resistance developing in vectors and malaria parasites, a changing environment and climate, and an increasing mobility of people travelling to and from malaria endemic areas within the country and across international borders [4,13,15].

The medical entomology surveys of the past showed that the distribution of malaria vectors and their malaria transmission capacity were confined to altitudes below 1,200 m above sea level (asl) in Nepal. Accordingly, the national malaria control programme only covers the area below 1,200 m [6,13,16-18]. The continuous effort of government and external development partners to control malaria in high endemic areas, with massive intervention programmes such as the distribution of insecticide treated bed nets and indoor residual spraying, has significantly reduced the total number of confirmed malaria cases over the years in Nepal [8]. Despite this achievement, malaria cases which had once been confined to the lowlands and forest areas are now already reported from highland areas of Nepal above 2,000 m [19]. However, medical entomology evidence is lacking to explain whether malaria vectors causing local transmission are established in high altitude habitats of Nepal or not.

Recent evidence suggests that the warming rate in higher altitudes and latitudes is faster than in the lowlands [20-22]. This is also supported by the analysis of climate data in Nepal [23-28]. The changing climate and other factors can thus create a conducive environment for the survival, development and breeding of mosquitoes in new areas. Many recent studies have predicted an impact of climate change on the geographical distribution of disease vectors [29-31] and an increasing epidemic potential of vector-borne diseases (VBDs; mainly malaria and dengue fever) in temperate regions and tropical highlands [32-35]. The altitudinal transect survey carried out for the preparation of the National Adaptation Programme of Actions to climate change (NAPA) in Nepal reports that mosquito nuisance has increased in the highlands over the last decade [36] and identifies VBDs as one of the major adaptation projects in the public health theme. Stimulated by these assumptions and events, we aimed to expand the current knowledge on the seasonal occurrence and altitudinal distribution of malaria vectors in eastern Nepal. We also aimed to document the seasonal occurrence and altitudinal distribution of the vectors of other major VBDs (dengue fever, lymphatic filariasis and Japanese encephalitis) if captured during the collection of *Anopheles* mosquitoes. However, we performed detailed statistical analyses of malaria vectors only because some important potential habitats of other VBD vectors such as discarded tires and other containers were not inspected, and many adults and larvae of *Culex quinquefasciatus* mosquitoes were not collected due to their large abundance in some areas. Therefore, the abundance data of these other disease vectors is not representative for inferential statistical analysis. Nevertheless, this study provides the first evidence of the altitudinal distribution of the vectors of malaria and other major mosquito-borne diseases in eastern Nepal.

## Methods

### Description of the study area and sampling techniques

**Figure 1:**
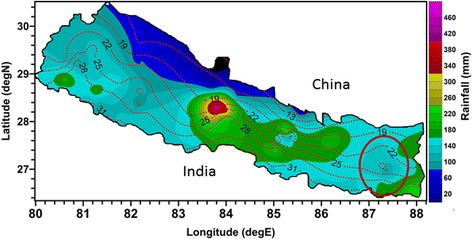
**Map of Nepal showing the study area (marked by red circle).** The map is a rough sketch and not to scale. Data for isohyets and isothermal contours was provided by the Department of Hydrology and Meteorology, Government of Nepal.

Surveys were carried out from September to October 2012 and April to May 2013 at fixed locations of three districts (Morang, Dhankuta and Terhathum) of eastern Nepal. The altitudes of the study sites vary from 70 m to 2,500 m asl. The study area was defined along an altitudinal transect and divided into lowland (<1,500 m asl) and highland parts (≥1,500 m asl) in terms of topography. In each locality, the collection spots faced different directions, and random collections were performed from each and every possible habitat. The map of study area is shown in Figure 1.

The average annual temperature of the study area ranges from about 31°C in the lowlands to about 22°C in the highlands. Similarly, the mean annual rainfall decreases from about 180 mm in the lowlands to less than 100 mm in the highlands. Both temperature and rainfall are favourable for the survival of *Anopheles* species, but it will be limited at least by the winter temperature which falls below 5°C. The altitude of eastern Nepal ranges from about 60 m to over 8,000 m asl. The highest peak mountains of the world above 8,000 m, Mt. Everest, Makalu and Kanchenjunga also lie in eastern Nepal where the Kanchenjunga and Makalu Himalayas stand just above our study area.

### Sampling of mosquitoes and identification

The eastern development region (equivalent to province) was purposively selected for this study from among the five development regions of Nepal. Subsequently the districts Morang, Dhankuta and Terathum were chosen for our study because they meet the criteria of covering high, moderate and low malaria transmission risk areas as well as ample altitudinal variation ranging from about 70 m to above 2,500 m. In consultation with District Public Health Offices and government medical entomologists, and by analyzing routine health surveillance data of malaria, 19 village development committees (VDC) were chosen for the entomological surveys in these districts. From each VDC at least 20 houses, natural outdoor shelters in their vicinity and potential breeding places were selected in each season (pre-monsoon and post-monsoon).

The entomological surveys were performed for one month (mid-September to mid-October) in 2012 and (mid-April to mid-May) in 2013. Adult mosquito sampling and the collection of immature stages was carried out as per WHO guidelines [37]. Mosquitoes resting indoors and outdoors were collected using flashlights and aspirators in the early morning (5:00–8:00 am). The rationale for making collections as early in the morning as possible was to avoid disturbance to mosquitoes by smoke from kitchens and by sun light. For collecting resting mosquitoes outdoors, natural shelters such as tree holes, stone cavities, erosion furrows, empty animal sheds and piles of firewood were inspected. Adult mosquitoes were also collected using a CDC light trap in human houses and animal shelters. The trap was set up in locations that were far away from competing light sources and hung up about 1.5 m above ground from dusk to dawn during one night per household or cattle shed. The light trap was kept turned on overnight. On the next morning the catch bag was removed and mosquitoes were immobilized with chloroform (ethanol stabilized; RFCL Limited, New Delhi) and identified using taxonomic keys and catalogues [3,38,39]. Small rain pools, seepage, streams, tree holes, water tanks ponds, rock pool, stone cave and artificial container such as discarded tires were surveyed for the presence of larvae, and collection were made by applying a standard sampling procedure described elsewhere [37,40]. Depending on the size of breeding habitat and the availability of larvae, three to ten samples were taken from each larval habitat. In streams, dipping was carried out at the edges and stream beds for a distance 300 m to 2,310 m depending on presence of larvae and access of stream water for sampling.

The identified mosquitoes were immediately transferred to cryovials and deep frozen in an MVE Doble 11 Dry Shipper using nitrogen vapor. Due to time and logistic constraints, mosquitoes were in some cases directly transferred to the dry shipper after identifying specimens up to genus level. Mosquito larvae were identified up to genus level using taxonomic and pictorial keys [3]. Then, the larvae were deep frozen in liquid nitrogen for species level identification using molecular genetic techniques (studies in progress).

### People’s perception data

As no previous altitudinal distribution data of mosquitoes was available for eastern Nepal, we assessed the perception of local people in the study areas about the distribution and occurrence of mosquitoes. For this we performed key informant interviews with those community leaders, social workers, teachers and health workers who had lived there for at least 30 years. The information collected mainly reflected the perception of people about the appearance of mosquitoes in terms of chronological order, associated causes for this, and perceived health risks.

### Environmental data

We collected the geographical coordinates, altitudes and environmental data of the study sites such as the number of animals kept in sheds, the number of persons who slept in the house in the night preceding the survey, etc. Mosquito collection resting sites were broadly categorized into human shelter (HS), animal shelter (AS), mixed shelter of animals and humans (MS) and natural outdoor shelter (NOS). The types of larval habitats and their characteristics such as description of habitat (i.e., temporary or permanent), water flow (i.e., stagnant or flowing), water condition (i.e., turbid, clean, polluted or turbid as well as polluted), water light (i.e., sunny or shaded) and water vegetation (i.e., with or without vegetation) were noted.

### Data analysis

The data were entered in Microsoft Excel 2010 spreadsheets and analyzed using R software [41]. The GPS coordinates of mosquito sampling points and malaria vector positive sites were projected onto maps with ArcGis software (ArcGis10, ESRI). The abundance data of mosquitoes were not normally distributed and showed a clumped distribution (variance > mean). Therefore, generalized linear models (GLM) were fitted assuming a negative binomial distribution and a log link function using the “Mass” package in R [42]. We fitted separate models for each species adult abundance of malaria vectors using season, topography (i.e., either lowland or highland) and resting sites of mosquitoes (i.e., animal shelter, human shelter, mixed human and animal shelter, natural outdoor shelters) as explanatory variables. Our models can be summarized as

$$ \mathrm{Adult}\ \mathrm{abundance} \sim \mathrm{Season} + \mathrm{Resting}\ \mathrm{site} + \mathrm{Topography} $$

We assessed the multicollinearity of the explanatory variables for each model using variance inflation factors (VIFs) which were less than 2.0. We used Akaike’s information criterion (AIC) to select the final model. Information on people’s perception of mosquitoes and mosquito-borne diseases was collected from eight qualitative interviews and manually summarized.

### Ethical approval

The conduct of this study was approved by the Ethical Review Board of the Nepal Health Research Council (NHRC), Government of Nepal. Oral informed consent was taken from the head of each household before starting the collection of mosquitoes either in houses or animal shelters. In cases where the household head disagreed, the house was dropped from the collection plan and the immediate next one was chosen for study. Written informed consent was taken from key informant interviewees.

## Results

### Mosquito species composition

A total of 2,538 adult mosquitoes belonging to the four genera *Aedes* (10%), *Anopheles* (55%), *Armigeres* (6%) and *Culex* (29%) were collected in both seasons (Table 1). Among the *Aedes* specimens (n = 245), the known dengue virus vectors in Nepal, *Aedes aegypti* and *Aedes albopictus*, constituted 3% and 4%, respectively. The principal vector of lymphatic filariasis, *C. quinquefasciatus*, and the Japanese encephalitis virus vector *Culex tritaeniorhynchus* accounted for 12% and 31% of the total collected *Culex* mosquitoes (n = 742). Similarly, the known malaria vectors in Nepal, *An. annularis*, *An. fluviatilis* and *An. maculatus* complex members, represented 16%, 9% and 10% of the total collected *Anopheles* mosquitoes (n = 1396). Hence, about 35% of the total *Anopheles* mosquitoes belonged to species that are known malaria vectors. The relative abundance of adult mosquitoes and their resting sites are presented in Table 1. Among the total *Anopheles* mosquitoes collected, 15% could not be identified up to species level under field conditions due to logistic and time constraints; these were directly preserved for molecular studies. About 23% of the collected *Anopheles* mosquitoes were assigned to *Anopheles* spp. other than the malaria vectors identified here based on morphological characters. Malaria vectors were found at all sampled seasons, topography and resting site (i.e., animal, human, mixed and natural outdoor shelters).

**Table 1:** **The seasonal abundance of adult mosquitoes and their resting sites**

**Mosquitoes species**	**Pre-monsoon**		**Post-monsoon**		**Both season**		**Resting sites**
	**Total collected (N)**	**Relative abundance (%)**	**Total collected (N)**	**Relative abundance (%)**	**Total collected (N)**	**Relative abundance (%)**	
*Aedes* (n = 245)							
*Aedes aegypti*	0	0	8	0.4	8	0.3	AS,NOS
*Aedes albopictus*	8	1.2	3	0.2	11	0.4	HS,HS
*Aedes* spp.	13	1.9	213	0.0	226	8.9	AS,HS,NOS
*Culex* (n = 742)							
*Culex quenquifasciatus*	87	12.7	4	0.2	91	3.6	HS,AS
*Culex tritaeniorhynchus*	11	1.6	217	11.7	228	9.0	AS,HS
Unidentified *Culex*	54	7.9	369	19.9	423	16.7	AS,HS,NOS
*Anopheles* (n = 1396)							
*Anopheles aconitus*	1	0.1	1	0.1	2	0.1	AS
*Anopheles nivipes*	3	0.4	2	0.1	5	0.2	AS
Anopheles tessellatus	10	1.5	1	0.1	11	0.4	AS
*Anopheles nigerrimus*	8	1.2	6	0.0	14	0.6	AS,HS
*Anopheles barbirostris*	10	1.5	9	0.5	19	0.7	AS,HS
*Anopheles splendidus*	13	1.9	16	0.9	29	1.1	AS,HS
*Anopheles sinensis*	23	3.4	14	0.8	37	1.5	AS,HS,NOS
*Anopheles culicifacies*	25	3.6	18	1.0	43	1.7	AS,HS,MS,NOS
*Anopheles pallidus*	32	4.7	66	0.0	98	3.9	AS
*Anopheles vagus*	86	12.6	22	0.0	108	4.3	AS,HS,MS
*Anopheles fluviatilis*	5	0.7	116	6.3	121	4.8	AS,HS,NOS
*Anopheles maculatus complex*	114	16.6	25	1.3	139	5.5	AS,HS,NOS
*Anopheles annularis*	27	3.9	201	10.8	228	9.0	AS, HS, MS
*Anopheles* spp.	74	10.8	258	13.9	332	13.1	AS
Unidentified *Anopheles*	0	0.0	210	11.3	210	8.3	HS
*Armigeres* (n = 155)							
*Armigeres* spp.	81	11.8	74	4.0	155	6.1	AS,HS,NOS
Total	685	100	1853	100	2538	100	

AS = Animal shelter, HS = Human shelter, MS = Mixed shelter of human and animal, NOS = Natural Outdoor Shelter.

Similarly, a total of 188 larvae of mosquitoes belonging to the three genera *Aedes* (49.5%), *Anopheles* (33%) and *Culex* (17.5%) were collected in both seasons (Table 1). We did not collect larvae of genus *Armigeres* because of their large abundance in most of the sampling habitats. The seasonal abundance and larval habitats of mosquitoes is presented in Table 2.

**Table 2:** **The seasonal abundance of mosquitoes larvae and their habitats**

**Characteristics**	**Larvae of collected mosquitoes genera**			
	***Aedes***	***Anopheles***	***Culex***	**Total**
**Pre-monsoon**				
Total collected	61	51	15	127
Breeding habitats	Stream, tree hole, water tanks, discarded tyre	Stream, seepage, water tanks, paddy fields	Stream, seepage, water tanks	
Description of breeding habitat	T/P	T/P	T/P	
Water condition	T/C	T/C	T/C/P	
Water flow	S/F	S/F	S/F	
Water light	Su/Sh	Su/Sh	Su/Sh	
Water vegetation	Both with/without	Both with/without	Both with/without	
**Post-monsoon**				
Total collected	32	11	18	61
Breeding habitats	Stream, tree hole, water tanks, discarded tyre	Stream, seepage, tree holes	Plastic drum, seepage, water tanks	
Description of breeding habitat	T/P	T/P	T	
Water condition	C	C	T/C/P	
Water flow	S	S/F	S	
Water light	Su	Su/Sh	Sh	
Water vegetation	Both with/without	Both with/without	Both with/without	
**Total collected larvae in both seasons**	93	62	33	188
Relative abundance (%)	49.5	33.0	17.5	100

T/P = Temporary/permanent C/T = Clean/Turbid C/T/P = Clear/Turbid/Polluted.

S/F = Stagnant/Flowing Su/Sh = Sunny/Shaded C = Clear S = Stagnant.

### Seasonal occurrence of vector mosquitoes

**Figure 2:**
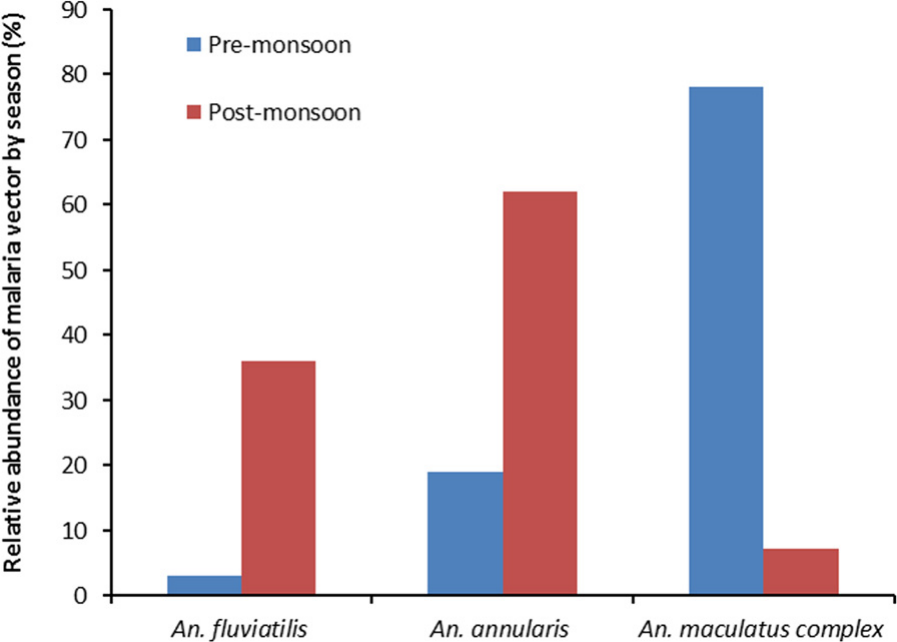
**Relative abundance of malaria vectors by season in eastern Nepal.**

All of the known malaria vectors in Nepal, *An. annularis*, *An. fluviatilis* and *An. maculatus* complex members, were recorded in both sampling seasons. The relative abundance of malaria vectors by season is shown in Figure 2. The dengue virus vector *A. aegypti* was only recorded in the post-monsoon season but other vectors (*A. albopictus*, *C. quinquefasciatus* and *C. tritaeniorhynchus*) were found in both seasons. Similarly, larva stage of three genera *Aedes*, *Anopheles* and *Culex* were found in both seasons (Table 2).

### Altitudinal distribution of vector mosquitoes

**Figure 3:**
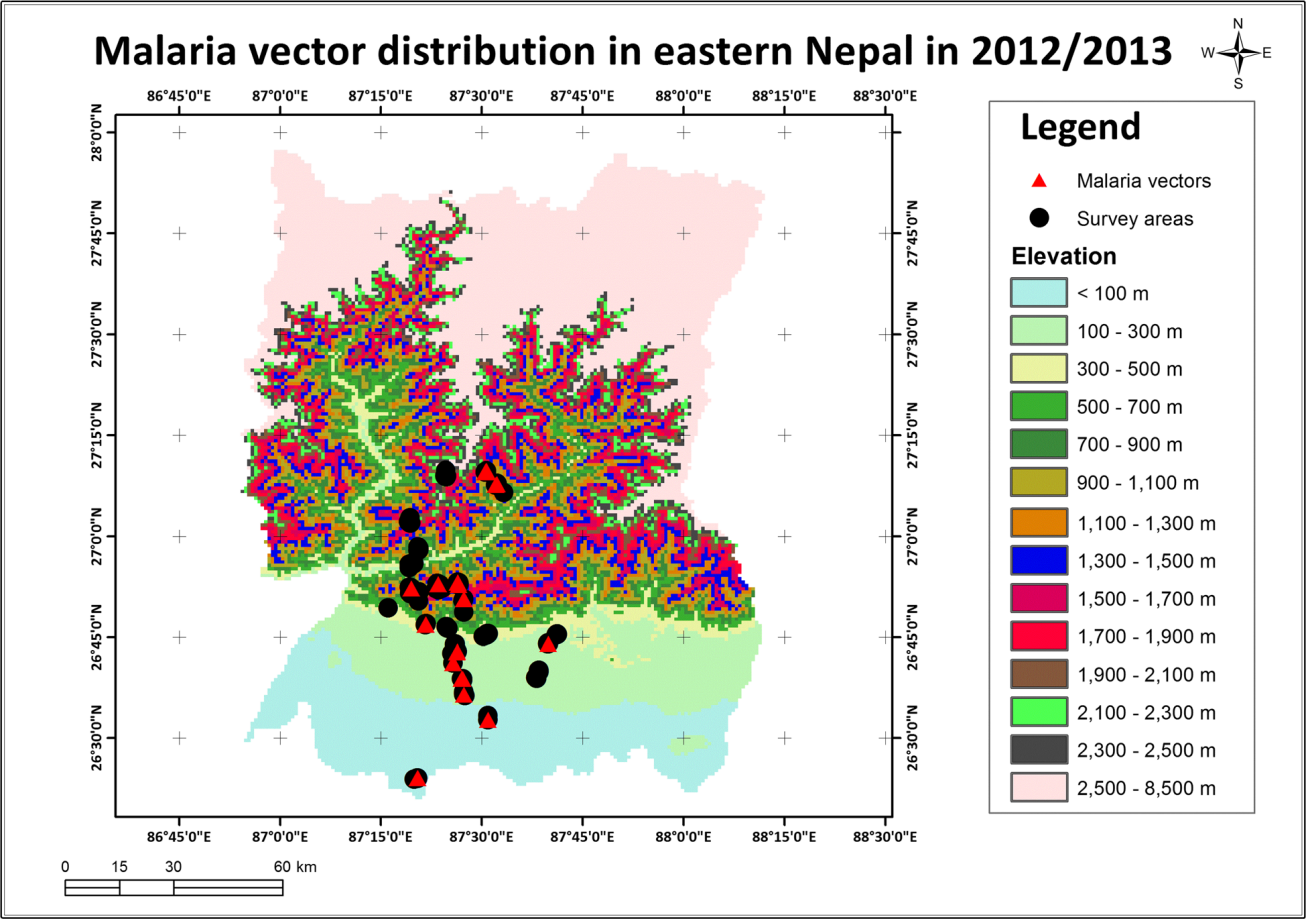
**Altitudinal distribution of malaria vectors in eastern Nepal, 2012/2013.** The altitudinal range surveyed extended from 70 to 2,500 m above sea level.

All three known malaria vector species were found throughout the studied altitude range up to 1,820 m asl (Figure 3). Similarly, other disease vectors *A. aegypti*, *A. albopictus*, *C. quinquefasciatus* and *C. tritaeniorhynchus* were also collected throughout the studied transect ranging from 70 to 2,000 m asl. We also collected *Anopheles*, *Culex* and *Aedes* larvae up to 2,310 m in our study area; their DNA-based species identification is in progress.

### Effect of season, topography and resting site on vector abundance

The effects of season, topography and resting site on the abundance of adult malaria vectors is summarized in Table 3. Only season had a significant effect on the mean abundance of *An. fluviatilis*. Compared to the post-monsoon (rainy) season, the abundance of *An. fluviatilis* was only 6% (95% CI = 0.01-0.31, P < 0.01) in the pre-monsoon (dry). The abundance of *An. fluviatilis* was 48 times higher in lowland (<1,500 m asl) compared to highland habitats (>1,500 m asl) (95% CI = 6.06-380.97, P = 0.06) although this difference was not statistically significant. Season, topography and resting site had significant effects on the mean abundance of *An. annularis*. The mean abundance of *An. annularis* was only 15% in the pre-monsoon compared to the post-monsoon season (95%CI =0.04-0.53, P < 0.01), only 23% in human shelters (95%CI = 0.06-0.82, P < 0.05) compared to animal shelters, and 4.25 times higher in lowland compared to highland habitats (95% CI = 1.09-16.62, P <0.05). Similarly, only season and resting site had significant effects on the mean abundance of *An. maculatus* complex members. The mean abundance of *An. maculatus* complex members was 3.50 times higher in pre-monsoon compared to post-monsoon (95%CI = 1.10-1.15, P < 0.05) and only 25% in human compared to animal shelters (95% CI =0.08-0.83, P < 0.05).

**Table 3:** **Effects of season, resting site and topography on the abundance of malaria vectors**

**Dependent variables**	**Explanatory variables**	**Coefficients**	**95% CI**
*An. fluviatilis*	(Intercept)	0.04	(0.01-0.27)***
	Season: Post-monsoon	1.00	1.00
	Pre-monsoon	0.06	(0.01-0.31)**
	Resting site: Animal shelter		
	Human shelter	0.28	(0.06-1.28)
	Mixed shelter	ND	ND
	Natural outdoor shelter	ND	ND
	Topography: Highland	1.00	1.00
	Lowland	48.04	(6.06-380.97)
*An. annularis*	(Intercept)	0.35	(0.09-1.38)
	Season: Post-monsoon	1.00	1.00
	Pre-monsoon	0.15	(0.04-0.53)**
	Resting site:: Animal shelter	1.00	1.00
	Human shelter	0.23	(0.06-0.82)*
	Mixed shelter	0.94	(0–853.43)
	Natural outdoor shelter	ND	ND
	Topography: Highland	1.00	1.00
	Lowland	4.25	(1.09-16.62)*
*An. maculatus* complex	(Intercept)	0.08	(0.02-0.30)***
	Season: Post-monsoon	1.00	1.00
	Pre-monsoon	3.50	(1.10-1.15)*
	Resting site: Animal shelter	1.00	1.00
	Human shelter	0.25	(0.08-0.83)*
	Mixed shelter	ND	ND
	Natural outdoor shelter	0.23	(0.02-3.02)
	Topography: Highland	1.00	1.00
	Lowland	2.64	(0.76-9.14)

Significant difference at P <0.05 (*), at P <0.01 (**) and at P <0.001 (***) (Two-tailed).

ND means not determined.

### People’s perception of mosquito occurrence and distribution

The local people of lowland areas did not perceive any change in the occurrence and distribution of mosquitoes in their communities. However, they reported that mosquito biting problems at night now had prolonged seasons including in winter whereas in the past this was limited mainly to the pre-monsoon and monsoon seasons (April-September). They also reported that people increasingly used bed nets at night and that malaria incidence had declined compared to the past. In contrast, people of highland areas perceived that mosquito nuisance had started in their communities as recently as 5–10 years ago and increasingly became a problem as mosquito bites started immediately after winter and lasted until the end of autumn. All participants univocally responded that the use of bed-nets would prevent mosquitoes-borne diseases. Participants of highland areas believed that mosquitoes had been carried to the highlands by trucks and buses and the creation of breeding places following the installation of water supply pipes in communities, domestic water storage and growing mosquito populations favoured by warming temperatures in the last years. They also believed that the replacement of biomass solid fuel by electricity promoted mosquito populations in households because electric lights attract mosquitoes whereas smoke from kerosene lamps and biomass fuels repels mosquitoes. In summary, community people perceived mosquito occurrence and distribution in the highlands to be a recent event which coincided with development and environmental changes including pronounced temperature increases in the latest decade. For example, a 60-year-old community leader in Dandabazar, Dhankuta (1,900 m asl), summarized his experience as follows: “*I have been living here for 40 years and a lot of development changes occurred over the years which are good as well as bad. For example, the development of residential areas and rural roads in the highlands has made our life easier, but at the same time mosquitoes which used to be found in the lowlands are now transported to the highlands and are established in our areas above 2,000 m altitude. This is primarily due to imported goods transported by buses and trucks from the lowlands and overnight stays of vehicles in the highlands. Unplanned urbanization as well as pig farming in densely populated settlements of the highlands have attracted and fostered larger mosquito populations. No matter if these mosquitoes cause diseases or not; every day we have to face mosquito bites, at least six months per year. No mosquito-borne diseases have ever been reported in our village but this may be due to the lack of diagnostic facilities in health posts and because seriously ill people are referred to the big hospitals in the lowlands. Most households in our village use bed nets at least during the six months from April to September. They prevent mosquito-borne diseases. I do not know about climate change but we have experienced a warming over the years and this has also produced larger mosquito populations in our areas”.*

## Discussion

This study provided information on the distribution of malaria vectors in eastern Nepal. The distribution of malaria vectors up to 1,820 m and the presence of *Anopheles* spp. larvae at 2,310 m asl are new records for eastern Nepal. All three known malaria vectors in this country, i.e., *An. fluviatilis*, *An. annularis* and *An. maculatus* complex members, were found to be distributed throughout the studied altitudinal range up to 1,820 m asl. Beside malaria vectors, mosquito species known to transmit the viruses causing dengue fever (*A. aegypti* and *A. albopictus*) and Japanese encephalitis (*C. tritaeniorhynchus*) as well as *Wuchereria bancrofti* microfilariae, the causative agents of lymphatic filariasis (*C. quinquefasciatus*), were encountered throughout the sampled altitudinal range from 70 to 2,000 m asl and probably (larvae pending molecular confirmation of species identification) above 2,300 m, which constitute altitudinal records for eastern Nepal. In the absence of previous entomological data or baseline evidence, we infer from qualitative data based on key informant interviews that these mosquito species recently established noticeable populations in the highlands of eastern Nepal.

Our results are consistent with the high-altitude *Anopheles* survey results of many published works. In western Nepal, the presence of *An. fluviatilis* and *An. willmori* along with their role in malaria transmission was demonstrated at high elevations (1,310 m and 1,980 m, respectively) in 1969 [43], but there has been no published evidence of malaria vectors in the highlands of eastern Nepal. In central Nepal, the malaria vectors *An. fluviatilis*, *An. annularis*, *An. maculatus* and other *Anopheles* species (*An. hycranus*, *An. splendidus*, *An. subpictus* and *An. lindesayi*) were recorded up to 1,700 m asl in highland valleys in 1960 [16]. Pant et al. [16] also reported that *An. hycranus* (79%) dominated in the highlands of central Nepal, followed in the post-monsoon season by *An. fluviatilis* (12%) and *An. annularis* (9%). However, in our study area in eastern Nepal we found a domination of *An. annularis* (16%) followed by *An. maculatus* complex members (10%) and *An. fluviatilis* (9%). Our findings are consistent with data from the highland Gum Valley of Mugu district in western Nepal where *An. fluviatilis* and *An. willmori*, a member of the *An. maculatus* complex, had been reported to be the dominant mosquitoes and vectors of malaria [43]. Furthermore, our findings on the distribution of *An. maculatus* complex members are similar to those from the western Himalayas in the Gharwal region of India except for *An. fluviatilis* and *An. annularis* which were reported only below 1,000 m in that area [44,45]. We could not record any *An. hycranus* in our study, and other studies have not reported this species after 1960 in Nepal, either [3,11,46]. Our findings on the distribution of dengue virus and lymphatic filariasis vectors in the highlands of eastern Nepal is consistent with recent reports on their distribution in central Nepal [47,48].

The findings of our study suggest that malaria and other disease vectors are already established in the highlands of Nepal posing a great health risk to mountain people who have no or only semi-immunity to malaria. In order to understand the effects of climate and other environmental change on the distribution of these malaria vectors and their potential for malaria transmission, models that integrate socio-economic, environmental and climatic variables are necessary. Nevertheless, in the absence of such studies we cannot ignore the contribution of the mobility of people, road development and an increasing access to means of transportation in accelerating distributional shifts of mosquitoes to higher altitudes. Interestingly, we exclusively found species in the highlands that are also found in the lowlands and no new species were recorded in the highlands, indicating that these species may have expanded their distributions from lowland to highland areas. However, in order to test this hypothesis molecular studies on the population genetics and historical area dynamics of these species are needed. An establishment of mosquitoes including known disease vectors has been reported in many studies from neighbouring countries of the Greater Himalayan region and Tibetan Plateau [49-51]. Malaria, Japanese encephalitis and lymphatic filariasis cases had previously been reported from all vector positive areas of the three districts.

The establishment of the dengue and chikungunya virus vectors *A. aegypti* and *A. albopictus* in the highlands of eastern Nepal in combination with the widespread belief of people that the use of bed nets is sufficient for preventing all mosquito-borne diseases indicates an elevated risk of chikungunya and dengue virus transmission because *A. aegypti* and *A. albopictus* are day-biting mosquitoes. These findings are consistent with those of a recent study that reported a low knowledge of dengue prevention and control in central Nepal [52]. A dengue fever outbreak was reported in the lowlands of neighbouring Jhapa district in 2012 [53] and a few cases of dengue fever from the hills of Dhankuta district in 2013 (unpublished data).

Both beneficial and adverse effects of development projects on malaria transmission have been reported in Nepal [18]. During our field work, we observed that an increasing access to water supply pipes in individual houses, the construction of ponds for fisheries, vegetable farming, road access in villages to vegetable marketing, a frequent movement of vehicles and micro-hydro power electricity gridlines in communities might have created more conducive environments for the passive and active dispersal of mosquitoes in the highlands of Nepal. We asked highland inhabitants about the prevalence of mosquitoes in their area, and these responded that mosquito problems had started in the village once the electricity line had been connected at home, and have existed 5–10 years overall. However, after a series of key informant interviews, we concluded that mosquito nuisance started 5–10 years ago following development work in the village and the problem of mosquito bites became prominent after the introduction of electricity, as electric light attracted mosquitoes in the evening. Previously, people used to light kerosene lamps and biomass fuel (wood) which acted as repellents to mosquitoes as has also been reported from India [54]. However, a review of the published literature shows no evidence that biomass smoke provides effective protection from mosquitoes and malaria [55].

The fact that malaria transmission has not been reported from higher altitudes in Nepal yet may be due to a lack of diagnostic facilities and poor recording and reporting systems as well as poor access to health services, in addition to the remoteness of the areas [4,6,56]. Other possible reasons include the zoophilic habits of *An. fluviatilis* which prefer animals over humans as prey in the highlands [16] and the use of bed nets while sleeping. During our entomological survey we also interviewed villagers about protective measures against mosquito bites, and the majority of people in households up to 2,000 m asl responded that they used bed nets throughout the year except in winter. According to the experience of community people, the density of mosquitoes starts to rise in the pre-monsoon and they disappear with the onset of winter. The perception of people on mosquitoes occurrence in the present study are consistent with results of previous studies [36,57,58].

This study was conducted only in the pre- and post-monsoon seasons and therefore does not take into account the overall seasonal variations of mosquito population dynamics. Hence, further longitudinal studies in this and additional regions are recommended taking into consideration all of the seasons and a broader altitudinal coverage of Nepal. Another important point to note here is that we were unable, for logistical constraints, to survey the steep intermediate altitudinal gradient between 500 and 1,000 m, nor areas above 2,300 m. Thus, our data cannot be extrapolated to these altitudes where totally different *Anopheles* species and mosquito communities might occur.

Our data revealing a present distribution of malaria vectors up to at least 1,820 m in eastern Nepal indicate that mountain people are at a particular risk because they lack immunity against malaria parasites. From a vector control point of view, indoor residual spraying will have little effect on the density of *An. fluviatilis* since these mostly hide in natural outdoor shelters. Spraying insecticides in natural outdoor shelters is impracticable because of its high cost and adverse effect on the environment. The ineffectiveness of indoor residual spraying on malaria vector density and malaria transmission has already been noted in Nepal [4,11,46,59]. This problem will be further aggravated by climate change which creates conducive environments for the development and breeding of mosquitoes and parasites in higher altitudes. Hence, the findings of this study will be useful for the Government of Nepal, external development partners and I/NGOs in their efforts to design and implement malaria control programs in higher altitude risk areas in order to achieve the ambitious goal of malaria elimination in Nepal by 2026.

## Conclusions

Based on the findings of our own studies in eastern Nepal, and published research evidence from central and western Nepal, malaria transmission in the higher altitudes of Nepal is possible because malaria vectors are already established in highland areas. Furthermore, infected people who are carriers or reservoirs of malaria parasites are introducing the latter to new areas due to the ever increasing movement of people to and from malaria endemic areas within the country and abroad. Accordingly, malaria cases have already started to be reported from the highlands of Nepal. However, they are likely grossly underreported because of the remoteness of these areas and the general belief that malaria transmission is not possible in places higher than 1,200 m asl in Nepal. Furthermore, the malaria control programme of the Government of Nepal is restricted to areas below 1,200 m. Thus, we conclude that malaria vectors are already established in higher altitudes of Nepal, and vector control programmes are urgently needed to protect the health of the people living in the mountains of this country.
